# Individual and contextual factors associated with mistimed and unwanted pregnancies among adolescent girls and young women in selected high fertility countries in sub-Saharan Africa: A multilevel mixed effects analysis

**DOI:** 10.1371/journal.pone.0241050

**Published:** 2020-10-22

**Authors:** Bright Opoku Ahinkorah

**Affiliations:** School of Public Health, Faculty of Health, University of Technology Sydney, Liverpool, Australia; University of Botswana, BOTSWANA

## Abstract

**Introduction:**

Unintended pregnancies are associated with a number of risk factors such as malnutrition, mental illness, unsafe abortion, maternal mortality and horizontal transmission of HIV to children. These risks are predominant among adolescent girls and young women compared to older women. This study examined the individual and contextual factors associated with unintended pregnancy among adolescent girls and young women in selected high fertility countries in sub-Saharan Africa.

**Materials and methods:**

Data for this study was obtained from recent Demographic and Health Surveys carried out between 2010 and 2018 in 10 countries in sub-Sahara Africa. The sample size for this study was made up of 6,791 adolescent girls and young women (aged 15–24), who were pregnant during the surveys and had complete responses on all the variables considered in the study. Unintended pregnancy was the outcome variable in this study. Descriptive and multilevel logistic regression analyses were performed and the fixed effect results of the multilevel logistic regression analysis were reported as adjusted odds ratios at 95% confidence interval.

**Results:**

Unintended pregnancy in the selected countries was 22.4%, with Angola, recording the highest prevalence of 46.6% while Gambia had the lowest prevalence of 10.2%. The likelihood of unintended pregnancy was high among adolescent girls and young women aged 15–19 [aOR = 1.48; 95% CI = 1.26–1.73], those with primary [aOR = 1.99; 95% CI = 1.69–2.33] and secondary/higher [aOR = 2.30; 95% CI = 1.90–2.78] levels of education, single (never married/separated/divorced/widowed) adolescent girls and young women [aOR = 9.23; 95% CI = 7.55–11.28] and those who were cohabiting [aOR = 2.53; 95% CI = 2.16–2.96]. The odds of unintended pregnancy also increased with increasing birth order, with adolescent girls and young women having three or more births more likely to have unintended pregnancies compared to those with one birth [aOR = 1.99; 95% CI = 1.59–2.48]. Adolescent girls and young women who had ever used contraceptives (modern or traditional), had higher odds of unintended pregnancies compared to those who had never used contraceptives [aOR = 1.32; 95% CI = 1.12–1.54]. Finally, adolescent girls and young women who belonged to the rich wealth quintile were more likely to have unintended pregnancy compared to those in the poor wealth quintile [1.28; 95% CI = 1.08–1.51].

**Conclusion:**

The study found that age, marital status, level of education, parity, use of contraceptives and wealth quintile are associated with unintended pregnancy among adolescent girls and young women in high fertility sub-Saharan African countries. These findings call for the need for government and non-governmental organisations in high fertility sub-Saharan African countries to restructure sexual and reproductive health services, taking into consideration these individual and contextual level characteristics of adolescent girls and young women.

## Introduction

Globally, 44% of mistimed and unwanted pregnancies (unintended pregnancies) were recorded from 2010 to 2014 and this translates into 62 unintended pregnancies per 1,000 women within 15–44 age period [1]. This marks a decline from 74 unintended pregnancies per 1,000 women aged 15–44 between 1990 and 1994. However, the reduction is uneven with high-income countries achieving 30% reduction whilst the low-and middle-income countries accomplished 16% reduction [1]. Almost 14 million unintended pregnancies are recorded every year and 44% are among adolescent girls and young women (AGYW) aged 15–24 years in sub-Saharan Africa (SSA) [2, 3]. The incidence of unintended pregnancies has also been found to be high in societies at each stage of fertility transition [4, 5].

In the early 1950s, fertility levels in Africa were high (6.5) and nearly stable, indicating that all societies in SSA were in the pre-transitional stage, with little deliberate effort to reduce fertility through the use of contraception or abortion. In 2000, there was a decline in fertility in SSA but only at a pace of 0.03 per year [6]. As at 2015, nearly all developing countries had entered the fertility transition. However, decline in SSA began later and has been slower, as fertility rates between 2010 to 2015 were still above five births per woman [6, 7].

A number of theories have been adduced to explain the causes of these fertility trends. Most of these theories conclude that the trends in fertility are partly influenced by a decline in couples’ desired family size [8–10]. Other theorists also attribute the decline in couples’ desired family size to the diffusion of new ideas about fertility limitation and the costs and benefits of children [11–15]. Bongaarts [16] also attribute the decline in fertility trends to increased use of contraceptive use by couples.

Unintended pregnancies have been found to be associated with numerous risk factors such as malnutrition, mental illness, unsafe abortion, maternal mortality, mental illness and horizontal transmission of HIV to children [17, 18]. In SSA, other risk factors such as stress, reduction in the quality of life and economic instability have been identified [19, 20]. Most of these risk factors are more pronounced in AGYW as most of them have been found to have higher rates of unsafe abortions, mental disorders, depression and mortality due to unintended pregnancies [21, 22]. This makes research on unintended pregnancies among this cohort of women, especially in SSA, very essential.

However, within the SSA region, most studies that have been conducted on unintended pregnancies are among all women of reproductive age (15–49) [2, 23–28]. Findings from these studies indicate that poor knowledge in contraceptive use, lack of access to contraceptives, contraceptive failure, sexual violence and lack of empowerment to make contraceptive decisions account for unintended pregnancies among women. Other predictors of unintended pregnancies in these studies were younger and older age, lower socio-economic status and ethnic and religious disparities. Among young women, Christofides, Jewkes [29], carried out a study on risk factors for unplanned and unwanted pregnancies in South Africa and found physical abuse and lower socioeconomic status as risk factors for unplanned and unwanted pregnancies.

Although, majority of these factors have been considered to predict unintended pregnancies among all women of reproductive age, the manner in which these factors contribute to unintended pregnancies may differ among AGYW, compared to adults. For instance, empirical studies [30–32] and theories [33, 34] have shown that AGYW face more challenges with access to contraception.

Hence, in order for declining fertility preferences to have their intended effect of reducing actual fertility, especially among AGYW, contraceptives have to be used effectively by sexually active AGYW to prevent unplanned pregnancies. However, high unmet need for contraception due to multiple barriers to contraception among AGYW [33, 34] account for the high rates of unintended pregnancies in SSA. Other scholars have also argued that most of the unintended pregnancies are related to sexual activities that occur outside marriage, which are more prevalent among AGYW, compared to adults [24, 26, 35, 36]. Even among AGYW, the risk of unintended pregnancy has been found to be high among those aged 15–19, compared to those aged 20–24 [29, 32, 37].

Apart from these individual-level factors, the society in which AGYW live in and the cultural norms and practices around them also act as contextual factors that play key roles in their decision to engage in behaviours that put them at risk of unintended pregnancies [38]. This is supported by Bandura’s Social Learning Theory, which emphasizes the mutual interaction between cognitive, behavioural and environmental determinants of human behaviour and postulates that people learn new behaviours by watching others in a social situation, absorbing and then emulating that behaviour [39]. The immediate social groups from whom people learn includes family, friends, teachers, neighbours and church groups. These social groups communicate attitudes, views and values that an individual can adopt and inculcate. They also emphasise appropriate social behavioural codes of conduct [40].

Based on the empirical and theoretical evidence that the factors that account for unintended pregnancies among AGYW and adults may differ, evidence that majority of studies on unintended pregnancies in SSA have been conducted among all women of reproductive age and the role of fertility transition in understanding unintended pregnancies, this study seeks to examine the individual and contextual factors associated with unintended pregnancy among AGYW in selected high fertility countries in SSA, using data from current nationally representative surveys in these countries.

## Materials and methods

### Data source

Data from current Demographic and Health Surveys (DHS) carried out between 2010 and 2018 in 10 SSA countries was used in this study. The selection of the 10 countries was based on a recent World Bank report that lists 10 SSA countries with fertility rates above 5.0 (more than the rate of 2.4 children per woman globally and the average rate of 4.8 in SSA). These countries and their fertility rates are as follows: Niger (7.2), Mali (5.9), Chad (5.8), Angola (5.6), Burundi (5.6), Uganda (5.5), Nigeria (5.4), Gambia (5.3), Burkina Faso (5.3) and Mozambique (5.1) [41]. For this study, the women’s files (IR) which contain the responses of women aged 15–49 were used. Fertility rates and other indicators of fertility transition of the selected countries obtained from their DHS have been provided in Table 1.

**Table 1:** List of countries and some indicators of fertility transition.

Country	Mean age at first sex	Mean age at first marriage	Mean age at first birth	Fertility rates
Angola	19	19	19	2.9
Burkina Faso	17	18	19	3.3
Burundi	14	20	21	2.6
Chad	20	16	18	3.9
Gambia	17	18	19	2.6
Mali	16	18	19	3.2
Mozambique	17	18	X	2.9
Niger	18	16	18	4.0
Nigeria	16	18	20	3.0
Uganda	16	18	19	3.1

Source: Author’s computation from DHS data.

X = No data in DHS.

DHS is a nationwide survey that is carried out across low-and middle-income countries every five-years [42] and dwells on a number of indicators such as unintended pregnancy. It evolved from World Fertility Surveys and Contraceptive Prevalence Surveys implemented in the 1970s and 1980s and originally collected comparable population-based data on fertility, contraception, maternal and child health and nutrition in developing countries [43]. DHS has been an essential data source on issues of reproductive health in low-and middle-income countries as it gathers data on a number of reproductive health issues such as marriage and sexual activity, fertility, fertility preferences and family planning [42]. Stratified dual-stage sampling approach was employed in selecting the sample for each survey. Previous studies have provided details of the sampling approach [42, 44]. The sample size for this study was made up of AGYW (aged 15–24), who were pregnant during the survey and had complete cases on all the variables of interest (N = 6791). Table 2 provides a detailed information on the survey country, year of survey and sample size for each country.

**Table 2:** Survey and sample size characteristics.

Country	Survey Year	Total adolescent girls and young women interviewed	Sample size by design^a^	Selected adolescent girls and young women sample^b^	% of completed responses
Angola	2015–16	6423	702	702	100%
Burkina Faso	2010	6592	679	674	99.3%
Burundi	2016–17	7218	408	406	99.5%
Chad	2014–15	6884	975	960	99.0%
Gambia	2013	4564	334	333	99.7%
Mali	2018	4116	494	494	100%
Mozambique	2015	2944	321	321	100%
Niger	2012	3869	539	531	98.5%
Nigeria	2018	15267	1444	1444	100%
Uganda	2016	8058	926	926	100%
Total	-	65935	6822	6791	99.5%

^a^Sample size by design are adolescent girls and young women aged 15–24, who were pregnant at the time of the study and responded to questions on pregnancy intention.

^b^Selected adolescent girls and young women sample are those with complete response for all variables considered.

### Outcome variable

“Pregnancy intention” was the outcome variable in this study. It was derived from a question in relation to women’s view on their current pregnancy. At the time of the survey, respondents were asked: "When you got pregnant, did you want to get pregnant at that time?” Three responses: ‘then’, ‘later’ and ‘not at all’ were generated from this question. In line with how unintended pregnancy has been defined in previous studies as “pregnancies that are either wanted earlier or later than occurred (mistimed) or not needed (unwanted)” [2, 23, 25, 45], a binary outcome was created from these three responses (then = 0 ‘planned’ later and not at all’ = 1 ‘unintended’).

### Explanatory variables

Ten explanatory variables were considered in this study and were grouped into individual and contextual-level variables. These variables were not determined a priori; but were selected based on their availability in the datasets, their theoretical relevance and practical significance with unintended pregnancy in previous studies [2, 23–28].

#### Individual level factors

The individual level factors were age, marital status, educational level, frequency of reading newspaper/magazine, frequency of listening to radio, frequency of watching television and parity. Age was coded as ‘15–19’ and ‘20–24’. Marital status was recoded into ‘married’, and ‘cohabiting’. Level of education was coded as ‘no formal education’ ‘primary’ and ‘secondary/higher’. Frequency of reading newspaper/magazine, listening to radio and watching television were each coded as ‘not at all’ ‘less than once a week’ and ‘at least once a week’. Parity was recoded as ‘zero birth’, ‘one birth’, ‘two births’, and three or more births’.

#### Contextual level factors

The contextual level variables were wealth quintile, sex of household head and place of residence. In DHS, wealth quintile is computed in the surveys’ database using Principal Component Analysis (PCA) [46] and is classified as poorest, poorer, middle, richer and richest. However, for the purpose of this study, wealth quintile was recoded as poor (poorer and poorest), middle and rich (richer and richest). Sex of household head was coded as ‘male’ and ‘female’ and place of residence was coded as ‘urban’ and rural’. The selection of wealth quintile, sex of household head and place of residence as household/community level variables was based on their categorization in the DHS [42] and their classification in previous studies [47–50].

### Statistical analyses

The data was analysed with STATA version 14.2 for windows. Three steps were followed to analyse the data. The first step involved the use of frequencies in presenting the prevalence of unintended pregnancy among young women for each of the 10 countries and the overall prevalence for all the countries using a bar chart. Secondly, the datasets for the 10 countries were appended and the distribution of the variables were first segregated into adolescent girls (15–19 years) and young women (20–24 years). This was done to appreciate how the independent variables are distributed among AGYW. This was followed by a distribution of unintended pregnancy across the individual and contextual level characteristics of all the respondents and the use of chi-square test of independence [χ^2^] to examine the associations between the independent variables and unintended pregnancy. Statistical significance at this level of analysis was determined using a p-value of 0.20 (see Table 3). Afterwards, a two-level multilevel binary logistic regression analysis was carried out to examine the individual, and contextual factors associated with unintended pregnancy. Per the two-level modelling in this study, women were nested within clusters to account for the variance in primary sampling units (PSUs). Clusters were regarded as random effect to take care of the unexplained variability at the contextual level [51].

**Table 3:** Distribution of unintended pregnancy among adolescent girls and young women by the explanatory variables (weighted).

	Adolescent girls (15–19 years)		Young women (20–24 years)		Unintended pregnancy among Adolescent girls and young women		
Variables	Frequency (n)	Percentage (%)	Frequency (n)	Percentage (%)	Frequency (n)	Percentage (%)	p-values
**Individual level variables**							
**Educational Level**							p<0.001
No formal Education	1,156	45.5	1,900	44.7	380	12.4	
Primary	860	33.9	1,178	27.7	614	30.2	
Secondary/Higher	525	20.7	1,171	27.6	529	31.2	
**Marital Status**							p<0.001
Single	427	16.8	330	7.8	455	60.0	
Cohabiting	437	17.2	882	20.8	430	32.6	
Married	1,677	66.0	3,037	71.5	638	13.5	
**Frequency of reading newspaper/magazine**							p<0.001
Not at all	2,349	92.4	3,816	89.8	1,351	21.9	
Less than once a week	108	4.3	242	5.7	90	25.6	
At least once a weak	84	3.3	192	4.5	83	29.9	
**Frequency of listening to radio**							p<0.01
Not at all	1,307	51.4	1,915	45.1	666	20.7	
Less than once a week	405	16.0	782	18.4	245	20.6	
At least once a weak	829	32.6	1,552	36.5	611	25.7	
**Frequency of watching television**							p<0.001
Not at all	1,776	69.9	2,803	65.9	910	19.9	
Less than once a week	238	9.4	452	10.6	157	23.0	
At least once a weak	526	20.7	995	23.4	454	29.8	
**Parity**							p = 0.137
Zero births	1,687	66.4	896	21.1	575	22.3	
One birth	701	27.6	1,401	33.0	452	21.5	
Two births	138	5.5	1,201	28.3	308	23.0	
Three or more births	14	0.6	752	17.7	188	24.5	
**Ever used contraceptives**							p<0.001
No	2,325	91.5	3,222	75.8	1,131	20.4	
Yes	215	8.5	1,030	24.2	392	31.5	
**Contextual level variables**							p<0.001
**Wealth quintile**							
Poor	1,230	48.4	1,796	42.3	577	19.0	
Middle	524	20.6	871	20.5	318	22.8	
Rich	788	31.0	1,583	37.3	631	26.7	
**Sex of household head**							p<0.001
Male	2,132	83.9	3,647	85.8	1,181	20.4	
Female	409	16.1	603	14.2	342	33.8	
**Place of residence**							
Urban	619	24.3	1,247	29.3	537	28.8	
Rural	1,922	75.7	3,003	70.7	986	20.0	

Four models were fitted, with the first model, known as the empty model showing the variance in the outcome variable attributed to the clustering of the PSUs without the explanatory variables. The second model had only the individual-level factors. Model 3 contained the contextual-level factors and the final model was the complete model that had the individual and contextual-level factors simultaneously (see Table 4). These models were fitted using a STATA command “melogit”. For comparing models, the Akaike’s Information Criterion (AIC) tests were used. The lowest AIC was used to determine the best fit model (see Table 5). For all models, the odds ratio and associated 95% confidence intervals (CIs) were presented. To check for correlation among the explanatory variables, a test for multicollinearity was done using the variance inflation factor (VIF) and the results showed no evidence of high collinearity among the explanatory variables (Mean VIF = 1.32, Maximum VIF = 1.58, and Minimum VIF = 1.11). Sample weight (v005/1,000,000) was applied to correct for over and under sampling while the SVY command was used to account for the complex survey design and generalizability of the findings.

**Table 4:** Multilevel logistic regression models showing fixed-effects results on factors associated with unintended pregnancy among adolescent girls and young women.

Variables	Model 0	Model 1	Model 2	Model 3
	aOR[95%CI	aOR[95%CI]	aOR[95%CI]	aOR[95%CI]
**Individual level variables**				
**Age**				
15–19		1.46***[1.25–1.70]		1.48***[1.26–1.73]
20–24		Ref		Ref
**Educational Level**				
No formal Education		Ref		Ref
Primary		1.98***[1.68–2.33]		1.99***[1.69–2.33]
Secondary/Higher		2.36***[1.96–2.84]		2.30***[1.90–2.78]
**Marital status**				
Single		8.99***[7.43–10.87]		9.23***[7.55–11.28]
Cohabiting		2.47***[2.11–2.89]		2.53***[2.16–2.96]
Married		Ref		Ref
**Frequency of reading newspaper/magazine**				
Not at all		Ref		Ref
Less than once a week		0.89[0.68–1.18]		0.87[0.66–1.15]
At least once a weak		0.82[0.59–1.13]		0.81[0.59–1.12]
**Frequency of listening to radio**				
Not at all		0.91[0.75–1.10]		0.92[0.76–1.11]
Less than once a week		Ref		Ref
At least once a weak		0.94[0.77–1.14]		0.94[0.77–1.14]
**Frequency of watching television**				
Not at all		Ref		Ref
Less than once a week		1.18[0.96–1.48]		1.15[0.92–1.43]
At least once a weak		0.98[0.82–1.15]		0.92[0.76–1.11]
**Parity**				
Zero births		0.66***[0.56–0.78]		0.65***[0.55–0.77]
One birth		Ref		Ref
Two births		1.41***[1.17–1.69]		1.42***[1.18–1.71]
Three or more births		1.95***[1.57–2.44]		1.99***[1.59–2.48]
**Ever used contraceptives**				
No		Ref		Ref
Yes		1.34***[1.14–1.57]		1.32***[1.12–1.54]
**Contextual level variables**				
**Wealth quintile**				
Poor			Ref	Ref
Middle			1.15[0.98–1.35]	1.12[0.95–1.33]
Rich			1.18*[1.02–1.37]	1.28*[1.08–1.51]
**Sex of household head**				
Male			Ref	Ref
Female			1.88***[1.62–2.18]	0.98[0.82–1.16]
**Place of residence**				
Urban			1.37***[1.19–1.59]	0.95[0.81–1.13]
Rural			Ref	Ref

Exponentiated coefficients; 95% confidence intervals in brackets; AOR adjusted Odds Ratios CI Confidence Interval

*p< 0.05

**p< 0.01

***p< 0.001.

**Table 5:** Multilevel logistic regression models showing random-effects results on factors associated with unintended pregnancy among adolescent girls and young women.

Random effects				
PSU variance (95% CI)	0.13(0.06–0.24)	0.06(0.12–0.26)	2.1(0.9–0.51)	2.1(0.9–5.3)
ICC	0.04	0.02	0.03	0.02
LR Test	χ^2^ = 11.96, p< 0.01	χ^2^ = 2.30, p<0.10	χ^2^ = 10.56, p< 0.01	χ^2^ = 2.31, p< 0.10
Wald χ^2^	Reference	829.6***	117.5***	832.2***
Model fitness				
Log-likelihood	-3175.8	-3114.7	-3549.6	-3110.7
AIC	7218.7	6263.4	7111.2	6263.3
N	6791	6791	6791	6791

PSU = Primary sampling unit; ICC = Intra-Class Correlation; LR Test = Likelihood ratio Test; AIC = Akaike’s Information Criterion; N = Sample size.

### Ethical approval

For this study, ethical permissions were not demanded since DHS datasets, already publicly available was used. Institutions that commissioned, funded, or managed the surveys were responsible for ethical procedures. ICF international as well as an Institutional Review Board (IRB) in respective country approved all the DHS surveys in line with the U.S. Department of Health and Human Services regulations for the protection of human subjects. The data for this study can be accessed on https://dhsprogram.com/data/available-datasets.cfm.

## Results

### Descriptive results

**Fig 1:**
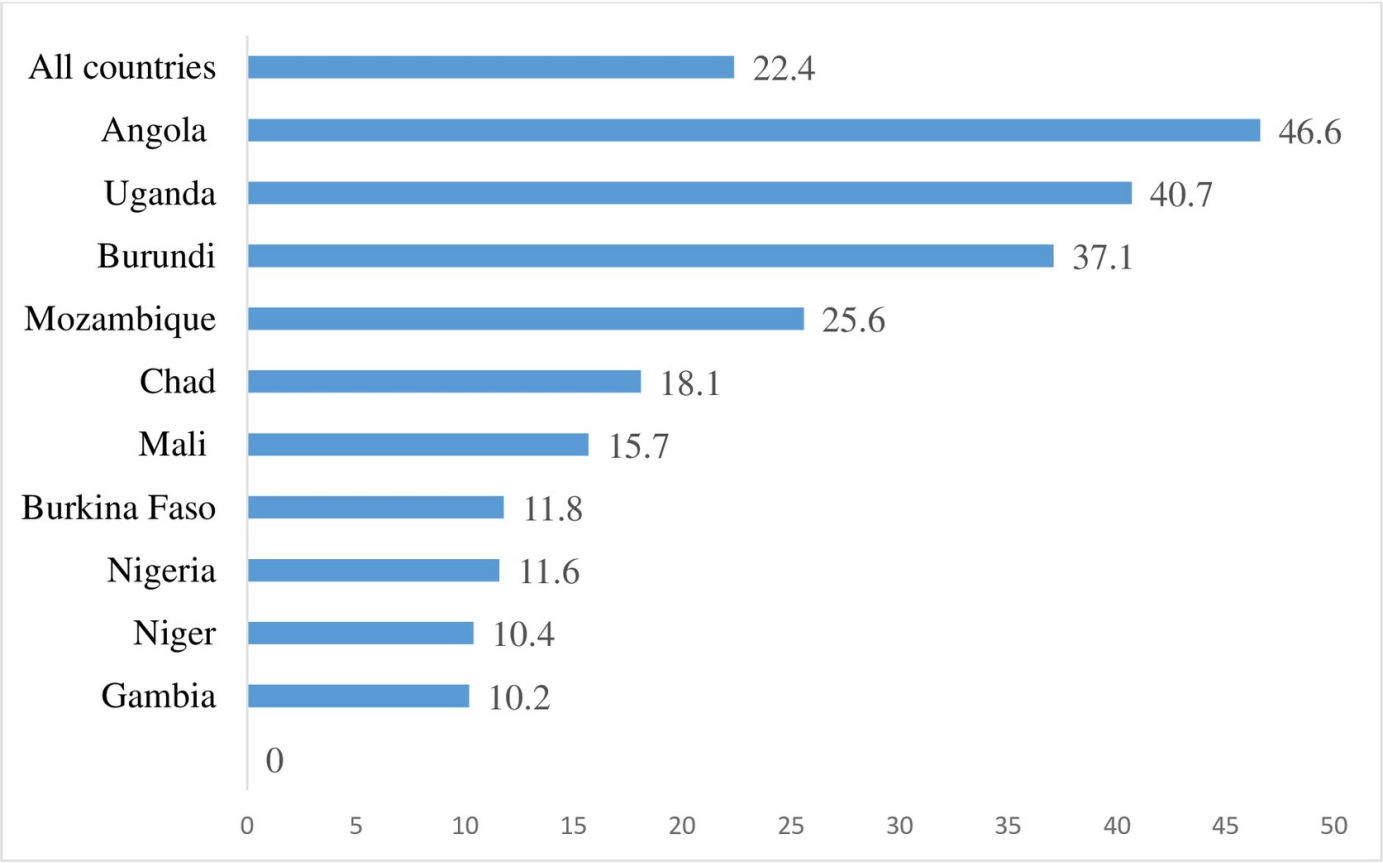
Prevalence of unintended pregnancy among adolescent girls and young women in selected high fertility sub-Saharan African countries.

Fig 1 indicates results of the prevalence of unintended pregnancy among AGYW in the 10 selected high fertility countries in sub-Saharan Africa. The prevalence of unintended pregnancy in all the countries was 22.4%, with Angola, recording the highest prevalence of 46.6% while Gambia had the lowest prevalence of 10.2%.

Table 3 shows results of the distribution of unintended pregnancy among AGYW in the selected high fertility countries in SSA by the explanatory variables. At the individual level, high prevalence of unintended pregnancy was found among AGYW aged 15–19 (24.3%), those with secondary/higher levels of education (31.2%) and those who were single (60.0%). Similarly, there was variations in unintended pregnancy with respect to the frequency of reading newspaper/magazine, frequency of listening to radio and frequency of watching television, parity and use of contraceptives.

With the contextual level factors, AGYW in the rich wealth quintile (26.7%), those in female-headed households (33.8%) and those who lived in urban areas (28.8%) had higher prevalence of unintended pregnancy. All the individual and contextual-level factors showed significant associations with unintended pregnancy at a p-value of 0.20 (see Table 3).

#### Multilevel logistic regression results

Model 3 of Table 4 shows the complete model of the fixed effect results of the associations between the individual and contextual level factors and unintended pregnancy among AGYW.

The likelihood of unintended pregnancy was high among AGYW aged 15–19 [aOR = 1.48; 95% CI = 1.26–1.73], those with primary [aOR = 1.99; 95% CI = 1.69–2.33] and secondary/higher [aOR = 2.30; 95% CI = 1.90–2.78] levels of education, single (never married/separated/divorced/widowed) AGYW [aOR = 9.23; 95% CI = 7.55–11.28] and those who were cohabiting [aOR = 2.53; 95% CI = 2.16–2.96]. The odds of unintended pregnancy also increased with increasing birth order, with AGYW having three or more births more likely to have unintended pregnancies compared to those with one birth [aOR = 1.99; 95% CI = 1.59–2.48]. AGYW who had ever used contraceptives (modern or traditional), had higher odds of unintended pregnancies compared to those who had never used contraceptives [aOR = 1.32; 95% CI = 1.12–1.54]. Finally, AGYW who belonged to the rich wealth quintile were more likely to have unintended pregnancy compared to those in the poor wealth quintile [1.28; 95% CI = 1.08–1.51].

Table 5 presents results on the random effects of the multilevel analysis. As shown in Table 5, in the empty model, there were substantial variations in the likelihood of unintended pregnancy across the clustering of the PSUs (σ2 = 0.13, 95% CI 0.06–0.24). The empty model showed that 4% of the total variance in unintended pregnancy was attributed to between-cluster variation of characteristics (ICC = 0.04). The between-cluster variations reduced by 2% in Model 1, from 4% in the empty model to 2% in the individual-level only model. From Model 1, the ICC increased to 3% (ICC = 0.03) in the contextual-level only model and further reduced to 2% in the complete model (Model 3), which had both the individual and contextual factors. This explains that the variations in the likelihood of unintended pregnancy could be attributed to the differences in the clustering at the primary sampling units (PSUs). Model fitness was determined using the AIC, with the smallest AIC value, indicating the best fit model. Hence, the final model, which contained all the explanatory variables was considered as the best fit model with an AIC value of 6263.3.

## Discussion

This study examined the individual and contextual factors associated with unintended pregnancy among AGYW in selected high fertility SSA countries. This is the first study that has looked at unintended pregnancy among AGYW in selected high fertility SSA countries. Unintended pregnancy was found to be 22.4% in the selected countries, with Angola having the highest prevalence of 46.6%. The study results further showed significant associations between marital status, parity, age, level of education, and use of contraceptives and unintended pregnancy. The prevalence of unintended pregnancy found in the current study is similar but lower than what has previously been found by Ameyaw, Budu (2), who identified a prevalence of 29% among women in SSA. The possible reason for the variations in findings could be explained by the differences in study population, sample size and study context. For instance, while the current study focused on AGYW, the study by Ameyaw, Budu (2) focused on all women of reproductive age. Again, whereas this study focused on the top 10 high fertility SSA countries, the study by Ameyaw, Budu (2) was done using 29 SSA countries. The high prevalence of unintended pregnancy among AGYW in Angola can be understood in the context of contraceptive use. This is because, Angola is a country that emerged from decades-long civil war [52] and it is expected that the fertility preferences of women and their desire for large family size should result in a reduction in unintended pregnancies. However, recent studies in the country have found high rates of unmet need for contraception among women in the country [53, 54] which can explain the high rates of unintended pregnancy. The high rates of unmet need has been explained by theorists to be associated with multiple barriers to contraception such as stigma, distance to healthcare facilities and cost of contraception, especially among AGYW [33, 34].

In this study, AGYW who had ever used contraceptives had higher odds of unintended pregnancy compared to those who had never used contraceptives. This finding may appear counterintuitive when considered within the context of contraceptive use helping to reduce unintended pregnancies as found in previous studies [32, 55]. However, the finding is expected considering evidence that majority of AGYW in SSA do not use modern contraceptives but rather traditional contraceptives for various reasons including stigma and discrimination by healthcare providers and fear of side effects [30–32]. Several studies have also argued that compared to modern methods of contraception, traditional contraceptives are not effective in preventing pregnancy [31, 56, 57]. There is the need for further studies on the trend and extent to which the use of traditional methods of contraception contribute to unintended pregnancy among AGYW in high fertility countries in SSA. A qualitative research should also be done to provide a more elaborate understanding of why contraceptive use increases the likelihood of unintended pregnancies.

Unintended pregnancy was high in AGYW aged 15–19, compared to those aged 20–24. Previous studies on unintended pregnancy in SSA corroborate the findings of the current study [23, 25, 27, 58]. Several authors have provided possible reasons for the inverse association between age and unintended pregnancy. For instance, Vázquez-Nava, Vázquez-Rodriguez [37], Christofides, Jewkes [29] and Grindlay, Dako-Gyeke [32] attribute high prevalence of unintended pregnancy among adolescents (15–19) to inadequate access to contraceptive services due to stigma and discrimination and risky sexual behaviour such as multiple sexual partnership and early sexual debut. Moreover, most adolescents in SSA have also been considered as a cohort of young women who often have low knowledge on contraceptives, inadequate access to information on sex due to socio-cultural norms and practices of society and lack the ability to negotiate for safer sex in sexual unions [59, 60]. The health belief model provides explanations to the likelihood of high unintended pregnancy among adolescents from the point of perceived risks [61] and a study comparing the risk perceptions of young adults and adolescents has suggested that younger people associate more risk with many behaviours [62].

AGYW who were single and those that had high parity had higher odds of unintended pregnancies. On the relationship between marital status and unintended pregnancy, there is consistency with the findings of previous studies [24, 26, 35, 36]. These authors argued that sexual activity for other reasons than childbearing is more frequent among unmarried women and this can increase the risk of unintended pregnancies. Others may also lack having communication on pregnancy with their partners, a phenomenon, which can put them at risk of unintended pregnancies. Frequent unprotected casual partners can also increase the risk of unintended pregnancy among single women. As AGYW give birth to more children, they are also more likely to consider subsequent pregnancies as unintended, as found in this study. This finding has been confirmed in previous studies as well [23, 25, 27, 58]. This finding is in line with the postulations of demographic theorists who attribute the decline in couples’ desired family size to the diffusion of new ideas about fertility limitation and the costs and benefits of children [11–15]. Hence, AGYW with higher parity may consider additional children as costs and this is likely to increase unintended pregnancies among them. By contrast, those with lower parity may desire for more children and see additional children as benefits and will be less likely to experience unintended pregnancies.

Unintended pregnancy was found to be high among AGYW with at least primary level of education and those with rich wealth quintile. Other similar findings have been obtained in previous studies [21, 28, 63]. For AGYW, the possible reason for this finding could be that those who have higher levels of education and higher wealth quintile are more likely to have higher desire to postpone childbearing in order to fulfil their educational and employment goals. For others who are not in school but working, they may see pregnancy that occurs in the course of their occupational career as mistimed or unwanted, especially where it has negative effect on their productivity [23]. The inverse relationship between socio-economic status and unintended pregnancies is supported by classical demographic theorists who attribute decline in fertility to increased schooling, urbanization, economic transformations and cultural modernization [8–10]. This implies that as modernization, urbanisation and higher levels of education improves the socio-economic status of AGYW, their fertility preferences shifts to the desire for fewer children and this can result in high levels of unintended pregnancies.

### Strength and limitations

The use of nationally representative datasets in this current study and the focus on AGYW in high fertility countries in SSA is a major strength in this study. Again, the large sample size and the adoption of well-laid procedures such as training of experienced field enumerators and the use of validated instruments in the DHS strengthen the validity of findings from the datasets. However, the use of cross-sectional design in the surveys makes it impossible to establish causality with respect to the findings, especially with the complex effect of variables such as marital status. Again, findings of the study can be generalized only to high fertility countries in SSA and not all SSA countries. Finally, pooling DHS data of Burkina Faso and Niger which were conducted much earlier than the rest of the countries may affect the findings.

## Conclusion

Based on the gap in literature in relation to the factors associated with unintended pregnancy among AGYW in high fertility SSA countries, this study examined the individual and contextual-level predictors of unintended pregnancy among AGYW in selected high fertility SSA countries. The study found that marital status, age, level of education, parity and use of contraceptives are associated with unintended pregnancy among AGYW in high fertility SSA countries. These findings call for the need for government and non-governmental organisations in high fertility SSA countries to restructure sexual and reproductive health services, taking into consideration these individual level characteristics of AGYW. Since modern contraceptives have been found to be effective in preventing unintended pregnancies, interventions aimed at enhancing access to modern contraceptives and encouraging their effective use among AGYW in high fertility SSA countries should be regarded as critical in reducing unintended pregnancy.
